# BCL6 degradation caused by the interaction with the C-terminus of pro-HB-EGF induces cyclin D2 expression in gastric cancers

**DOI:** 10.1038/sj.bjc.6605010

**Published:** 2009-03-31

**Authors:** Y Hirata, N Ogasawara, M Sasaki, T Mizushima, T Shimura, T Mizoshita, Y Mori, E Kubota, T Wada, S Tanida, H Kataoka, T Kamiya, S Higashiyama, T Joh

**Affiliations:** 1 Department of Gastroenterology and Metabolism, Nagoya City University Graduate School of Medical Sciences, 1-Kawasumi, Mizuho-cho, Mizuho-ku, Nagoya 467-8601, Japan; 2 Department of Biochemistry and Molecular Genetics, Ehime University Graduate School of Medicine, Shitsukawa, To-on, Ehime 791-0295, Japan

**Keywords:** BCL6, HB-EGF-CTF, cyclin D2, gastric cancer

## Abstract

BCL6 is a transcriptional repressor that has important functions in lymphocyte differentiation and lymphomagenesis, but there have been no reports of BCL6 expression in gastric cancers. In the present study, we investigated the BCL6 function in gastric cancers. Treatment with TPA resulted in BCL6 degradation and cyclin D2 upregulation. This phenomenon was inhibited by the suppression of the nuclear translocation of HB-EGF-CTF (C-terminal fragment of pro-HB-EGF). The HB-EGF-CTF nuclear translocation leads to the interaction of BCL6 with HB-EGF-CTF and the nuclear export of BCL6, and after that BCL6 degradation was mediated by ubiquitin/proteasome pathway. Real-time RT–PCR and siRNA targeting BCL6 revealed that BCL6 suppresses cyclin D2 expression. Our data indicate that BCL6 interacts with nuclear-translocated HB-EGF-CTF and that the nuclear export and degradation of BCL6 induces cyclin D2 upregulation. We performed immunohistochemical analyses of BCL6, HB-EGF and cyclin D2 in human gastric cancers. The inverse correlation between BCL6 and cyclin D2 was also found in HB-EGF-positive human gastric cancers. BCL6 degradation caused by the HB-EGF-CTF also might induce cyclin D2 expression in human gastric cancers. Inhibition of HB-EGF-CTF nuclear translocation and maintenance of BCL6 function are important for the regulation of gastric cancer progression.

BCL6 is a proto-oncogene located on chromosome 3q27 that encodes a transcriptional repressor (Baron *et al*, 1993; Kerckaert *et al*, 1993; Ye *et al*, 1993; Miki *et al*, 1994). BCL6 protein is a 92- to 98-kDa nuclear phosphoprotein that is produced at low levels in multiple tissues and is expressed at high levels exclusively in germinal centre B cells (Cattoretti *et al*, 1995; Onizuka *et al*, 1995). The formation of germinal centres and the development of T-cell-dependent humoral immune responses require BCL6 expression (Dent *et al*, 1997; Ye *et al*, 1997). BCL6 has an important function in germinal centre formation and the regulation of lymphocyte function, differentiation, and survival. High levels of BCL6 gene expression in non-Hodgkin's lymphomas have a favourable prognostic value (Alizadeh *et al*, 2000; Lossos *et al*, 2001). BCL6 contains an N-terminal BTB/POZ domain and six Kruppel-type (C_2_H_2_) zinc-finger (ZnF) motifs in the C terminus (Dhordain *et al*, 1995). The BTB/POZ domain displays a conserved protein–protein interaction motif and is required for the repressive activity of the protein (Jardin *et al*, 2007). The BTB/POZ domain interacts directly with the silencing mediator of retinoic acid and thyroid hormone receptor corepressor (or its relative N-CoR) (Dhordain *et al*, 1995, 1997). The ZnF motifs of BCL6 bind to DNA in a sequence-specific manner, leading to the transcriptional repression of target genes. The highest affinity site has a core sequence of 9 bp (TTCCT(A/C)GAA), and one of the target genes is cyclin D2 (Dent *et al*, 2002; Jardin *et al*, 2007). Thus, BCL6 is implicated in cell-cycle control by repressing cyclin D2. Recently, Logarajah *et al* (2003) and Bos *et al* (2003) reported that BCL6 has an important function in breast cancer. However, to our knowledge, there have been no reports of BCL6 in gastric cancer.

Heparin-binding epidermal growth factor-like growth factor (HB-EGF), a member of the EGF family, is synthesised as a 20- to 30-kDa type I transmembrane precursor (pro-HB-EGF) (Higashiyama *et al*, 1991, 1992). Ectodomain shedding is the process in which the pro-HB-EGF molecule is proteolytically cleaved by a disintegrin and metalloprotease (ADAM) 9, 12, 10, or 17 to release a soluble form of HB-EGF (Izumi *et al*, 1998; Asakura *et al*, 2002; Lemjabbar and Basbaum, 2002; Sunnarborg *et al*, 2002; Yan *et al*, 2002). Ectodomain shedding yields an N-terminal soluble ligand for the EGF receptor (HB-EGF) and a C-terminal fragment (HB-EGF-CTF) consisting of the transmembrane and cytoplasmic domains. The C-terminal fragment of HB-EGF is translocated from the cytoplasm to the nucleus and binds to a member of the BTB/POZ ZnF family, such as promyelocytic leukaemia zinc finger (PLZF) or BCL6 (Nanba *et al*, 2003; Kinugasa *et al*, 2007). This interaction reverses the transcriptional repression of PLZF or BCL6.

Gastric cancer is the second most common cancer in the world, accounting for ∼9% of all malignancies worldwide (Hohenberger and Gretschel, 2003). Increased expression and activation of EGFR and its ligands are causally associated with the progression of gastric cancer and poor prognosis (Barnard *et al*, 1995; D’Agnano *et al*, 1995). However, the exact mechanisms of HB-EGF-CTF activation within the gastric cancer environment are not fully understood.

In the present study, we investigated the interaction of BCL6 with HB-EGF-CTF and the mechanism of cyclin D2 expression by this interaction in gastric cancer cell lines, which express endogenous HB-EGF, BCL6, and cyclin D2. We also immunohistochemically studied 100 surgical specimens of advanced gastric cancers to analyse the expression of HB-EGF, BCL6, and cyclin D2. Our data indicate that BCL6 interacts with HB-EGF-CTF and that this interaction might induce the cyclin D2 expression in human gastric cancer.

## Materials and methods

### Materials

We purchased the following antibodies: rabbit anti-BCL6 monoclonal antibody (EP529Y; Epitomics, Burlingame, CA, USA); rabbit anti-cyclin D2 polyclonal antibody (M-20: sc-593; Santa Cruz Biotechnology, Santa Cruz, CA, USA); mouse anti-*β*-actin monoclonal antibody (Sigma-Aldrich, Tokyo, Japan); goat anti-HB-EGF polyclonal antibody (PX04; R&D Systems, Minneapolis, MN, USA); mouse anti-phospho-EGFR monoclonal antibody (9H2) and sheep anti-EGFR polyclonal antibody (Upstate, Lake Placid, NY, USA); and mouse anti-ubiquitin monoclonal antibody (P4D1), rabbit phospho-p44/42 MAP (mitogen-activated protein) kinase (Thr202/Tyr204) polyclonal antibody (D13.14.4E), rabbit p44/42 MAP kinase (MAPK) polyclonal antibody, and horseradish peroxidase-conjugated anti-mouse IgG antibody (Cell Signaling Technology, Beverly, MA, USA). The rabbit anti-HB-EGF-CTF polyclonal antibody and metalloproteinase inhibitor, KB-R7785, were kind gifts from Dr Higashiyama (Ehime University, Ehime, Japan). We obtained 12-0-tetradecanoylphorbor-13-acetate (TPA) and MEK 1/2 inhibitor U0126 from Cell Signaling Technology. The EGFR tyrosine kinase inhibitor AG1478 was purchased from Calbiochem (La Jolla, CA, USA).

### Cell culture

Human gastric cancer cells from the MKN45 cell line (Japanese Cancer Research Bank, No. 0254) were seeded in 6 cm dishes at a density of 2 × 10^6^ cells per dish and cultured for 48 h with RPMI-1640 (Sigma Chemical Co., St Louis, MO, USA) supplemented with 10% fetal calf serum and 1% ampicillin and streptomycin in 5% CO_2_. Human colon cancer cells HT29 were obtained from the American Type Culture Collection (Rockville, MD, USA) and human oesophageal cancer cells T.T were obtained from Health Science Research Resources Bank (Osaka, Japan). Both HT29 and T.T cells were cultured with Dulbecco's modified Eagle's medium (Sigma Chemical Co.) supplemented with 10% fetal calf serum and 1% ampicillin and streptomycin in 5% CO_2_.

### Western blotting

Subconfluent MKN45 cells were switched to serum-free medium for 24 h and then treated with TPA for the indicated times. Metalloproteinase inhibitor KB-R7785, EGFR tyrosine kinase inhibitor AG1478 and MEK 1/2 inhibitor U0126 were added to the cell cultures for 60 min before stimuli. Cells were washed with PBS (−) and subsequently dissolved in 1 × cell lysis buffer (Cell Signaling Technology) containing 20 mmol l^−1^ Tris-HCl (pH 7.5), 150 mmol l^−1^ NaCl, 1 mmol l^−1^ Na_2_EDTA, 1 mmol l^−1^ EGTA, 1% Triton, 2.5 mmol l^−1^ sodium pyrophosphate, 1 mmol l^−1^ *β*-glycerophosphate, 1 mmol l^−1^ Na_3_VO_4_, and 1 *μ*g ml^−1^ leupeptin. After disruption using a Bioruptor sonicator (Cosmo Bio, Tokyo, Japan) for 15 s, lysates were centrifuged at 15 000 r.p.m. for 10 min at 4°C. Each sample was normalised on an equal protein concentration by using a protein assay kit (Bio-Rad Laboratories, Hercules, CA, USA). An equal quantity of 2 × sodium dodecyl sulphate–polyacrylamide gel electrophoresis (SDS–PAGE) sample buffer (0.5 mol l^−1^ Tris-HCl (pH 7.2), 1% SDS, 100 mmol l^−1^ *β*-mercaptoethanol, and 0.01% bromophenol blue) was added to each sample, and then samples were boiled for 5 min at 100°C. Aliquots of boiled sample were fractioned on 7.5, 10, or 12.5% SDS–PAGE and then transferred to nitrocellulose membranes (Amersham Pharmacia Biotech, Buckinghamshire, England). The membranes were blocked with 5% skimmed milk in PBS (−) for 1 h at room temperature. The membranes were incubated with an appropriate antibody overnight at 4°C and then washed with 0.05% Tween 20 in PBS (−) three times at 5 min intervals. The membranes were incubated with an appropriate secondary antibody for 1 h at room temperature, followed by three washes with 0.05% Tween 20 in PBS (−) at 5 min intervals. Immunoreactive proteins were visualised by using the ECL Plus western blotting detection system (Amersham Biosciences, Buckinghamshire, England). Filters were stripped and reprobed with monoclonal *β*-actin antibody (Abcam Plc., Cambridge, MA, USA) as an internal control.

### siRNA

The siRNAs for the knockdown of BCL6 (siBCL6, CUGCGUCAUGCUUGUGUUAUA) and a scrambled siRNA (GCGCGCUUUGUAGGAUUCG), used as a negative control, were designed by and obtained from Qiagen (Tokyo, Japan). The scrambled sequence did not correspond to any mammalian sequence in the NCBI database and was used as a control. MKN45 cells were transfected with 20 *μ*mol l^−1^ siRNA by using Lipofectamine reagent (Invitrogen, Carlsbad, CA, USA) according to the manufacturer’s instructions. Assays were performed 2 days after MKN45 cells were transfected.

### Real-time RT–PCR

Total RNA was isolated from cells by using TRIzol reagent (Invitrogen). First-strand cDNA was synthesised by using Superscript II (Invitrogen) according to the manufacturer’s instructions. Primers for human cyclin D2 and control human glyceraldehyde-3-phosphate dehydrogenase (GAPDH) were obtained from Applied Biosystems (Tokyo, Japan; cyclin D2, Hs0027041_m1; GAPDH, 4310884E). Real-time RT–PCR was carried out on the ABI 7500 Fast Real-Time PCR system (Applied Biosystems). Each experiment was carried out in a 20 *μ*l reaction volume containing 18 *μ*l TaqMan Fast Universal PCR Master Mix (Applied Biosystems), 1 *μ*l cDNA, and 1 *μ*l primers. A uniform amplification of the products was rechecked by analysing the melting curves of the amplified products. All reactions were carried out in triplicate to assess reproducibility.

### Immunofluorescence microscopy

Human gastric cancer cells (MKN45) in a subconfluent state were switched to serum-free medium for 24 h and then treated with 200 nmol l^−1^ TPA at the indicated times, and the subcellular localisation of HB-EGF-CTF and BCL6 was analysed by immunofluorescence study. Cells were fixed with ethanol and acetone. Incubation with primary antibodies was performed in a solution of PBS containing 0.1% milk at room temperature. Then, sections were incubated with the appropriate secondary antibodies and all sections were counterstained with 4′,6-diamidino-2-phenylindole (DAPI; Kirkegaard and Perry Laboratories, Gaithersburg, MD, USA). Images were obtained with an Eclipse 80i fluorescence microscope (Nikon, Tokyo, Japan).

### Immunoprecipitation

Cells treated with TPA were lysed with 1 ml of lysis buffer. After centrifugation of the lysates at 15 000 r.p.m. for 20 min, supernatants were collected and incubated with 1 *μ*g of anti-HB-EGF-CTF antibody or anti-BCL6 antibody for 2 h at 4°C with end-over-end rotation. Next, 20 *μ*l of protein G Sepharose beads (50% suspension; Amersham Pharmacia Biotech) was added to each lysate/antibody mixture, followed by incubation for 2 h at 4°C with end-over-end rotation. The mixtures were then centrifuged, and the protein G Sepharose beads were washed three times with lysis buffer, resuspended in 15 *μ*l of 2 × SDS–PAGE sample buffer and boiled for 3 min. The bound proteins were analysed by western blotting using an anti-BCL6 antibody or anti-ubiquitin antibody.

### Samples and tissue collection

We performed immunohistochemical staining of tissue samples from 100 cases of advanced gastric cancer that were surgically resected at Nagoya City University Hospital between 1998 and 2004. All specimens were fixed in 10% buffered formalin. Gastric cancers with adjacent non-neoplastic mucosa were cut serially into 4 mm slices in parallel with the lesser curvature. The slices were embedded in paraffin and stained with hematoxylin and eosin for histological examination.

### Immunohistochemistry

Immunohistochemical staining was performed with antibodies against the following antigens: BCL6 (EP529Y, dilution 1 : 100; Epitomics), cyclin D2 (M-20: sc-593, dilution 1 : 250; Santa Cruz Biotechnology), and HB-EGF (PX04, dilution 1 : 50; R&D Systems). The procedure was performed with the appropriate positive and negative controls. The immunohistochemical techniques were performed as previously described (Takenaka *et al*, 2007). Briefly, 4 *μ*m-thick consecutive sections were deparaffinised and hydrated through a graded series of alcohols. After inhibition of endogenous peroxidase activity by immersion in 3% H_2_O_2_/methanol solution, antigen retrieval was achieved by heating the samples in 10 mM citrate buffer (pH 6.0) in a microwave oven for 10 min at 98°C. Then, sections were incubated with primary antibodies. After thorough washing in PBS (−), the samples were incubated with biotinylated secondary antibodies and then with avidin-biotin horseradish peroxidase complexes (Vectastain Elite ABC kit; Vector Laboratories Inc., Burlingame, CA, USA). Finally, immune complexes were visualised by incubation with 0.01% H_2_O_2_ and 0.05% 3,3′-diaminobenzidine tetrachloride. Nuclear counterstaining was accomplished with Mayer’s hematoxylin.

Two independent researchers (YH and NO) judged the histology and immunohistochemical staining of the HB-EGF, BCL6, and cyclin D2. To investigate the relationship between HB-EGF, BCL6, and cyclin D2 expression, we analysed immunohistochemical staining of HB-EGF, BCL6, and cyclin D2 area by area. The localisations of BCL6 were heterogeneous in the same cancer tissues. However, we found 100 homogeneously stained regions >1 cm in diameter. Only nuclear immunostainings were considered to be positive for the evaluation of BCL6 and cyclin D2 staining, whereas nuclear and cytoplasmic immunostainings were considered to be positive for HB-EGF evaluation. Reactivity for HB-EGF, BCL6, and cyclin D2 was scored according to the percentage of positively stained tumour cells in the section areas on a three-point scale: score 0 (−),<10%; score 1 (+), 10–33%; score 2 (++), 34–66%; score 3 (+++), 67–100%. The expression was considered to be positive (+) with a score of 1 or higher (Mizoshita *et al*, 2003). When less than 10% of tumour cells were stained, immunostaining was considered to be negative.

### Statistical analysis

The data were analysed by Welch's *t*-test and *χ*^2^-test, as appropriate. *P*-values <0.05 were considered statistically significant.

## Results

### TPA-dependent ectodomain shedding induces phosphorylation of both EGFR and MAPK in MKN45 cells

**Figure 1:**
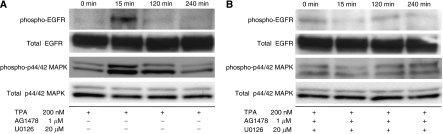
12-0-Tetradecanoylphorbor-13-acetate (TPA)-dependent ectodomain shedding induces phosphorylation of both EGFR and MAPK in MKN45 cells. (**A**) MKN45 cells were treated with 200 nM TPA for 0, 15, 120, and 240 min respectively. The endogenous phospho-EGFR and phospho-p44/42 MAPK, along with total EGFR and total p44/42 MAPK expression levels, were analysed by western blotting. (**B**) Before the treatment of MKN45 cells with 200 nM TPA for indicated periods, the cells were preincubated with 1 *μ*M AG1478 (an EGFR tyrosine kinase inhibitor) and 20 *μ*M U0126 (an MEK 1/2 inhibitor) for 60 min. The endogenous phospho-EGFR and phospho-p44/42 MAPK, along with total EGFR and total p44/42 MAPK expression levels, were analysed by western blotting.

We first examined whether TPA treatment induces phosphorylation of both EGFR and MAPK in MKN45 cells. 12-0-Tetradecanoylphorbor-13-acetate was used to induce cleavage of pro-HB-EGF by the activation of ADAM12. Cells were treated with 200 nM TPA for the indicated periods, after which the endogenous phospho-EGFR and phospho-p44/42 MAPK, along with total EGFR and total p44/42 MAPK expression levels, were analysed by western blotting. Treatment with TPA resulted in phosphorylation of both EGFR and p44/42 MAPK in MKN45 cells (Figure 1A). However, when 1 *μ*M AG1478, an EGFR tyrosine kinase inhibitor, and 20 *μ*M U0126, an MEK 1/2 inhibitor, were added to inhibit the EGFR signalling pathway before treatment with 200 nM TPA, phosphorylation of both EGFR and p44/42 MAPK was inhibited despite the TPA treatment in MKN45 cells (Figure 1B). Thus, these data indicated that TPA-dependent ectodomain shedding induced phosphorylation of both EGFR and MAPK, and AG1478 and U0126 inhibited the EGFR signalling pathway.

### BCL6 expression is decreased and cyclin D2 expression is increased following pro-HB-EGF shedding in MKN45 cells

**Figure 2:**
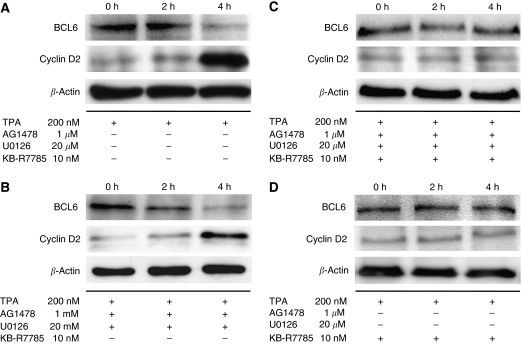
Nuclear translocation of HB-EGF-CTF induces degradation of BCL6 and upregulation of cyclin D2 expression. (**A**) MKN45 cells were treated with 200 nM TPA for 2 or 4 h. The levels of BCL6 and cyclin D2 protein were determined by western blotting with anti-BCL6 and anti-cyclin D2 antibody. (**B**) Before the treatment of MKN45 cells with 200 nM TPA for 2 or 4 h, the cells were preincubated with 1 *μ*M AG1478 and 20 *μ*M U0126 for 60 min. The levels of BCL6 and cyclin D2 protein were analysed by western blotting. (**C**) Before TPA stimuli, 10 *μ*M KB-R7785 (a metalloproteinase inhibitor) with both 1 *μ*M AG1478 and 20 *μ*M U0126 was added to the cells for 60 min. Then, MKN45 cells were treated with 200 nM TPA for 2 or 4 h. The levels of BCL6 and cyclin D2 protein were determined by western blotting. (**D**) Before TPA stimuli, 10 *μ*M KB-R7785 alone was added to the cells for 60 min. Then, MKN45 cells were treated with 200 nM TPA for 2 or 4 h. The levels of BCL6 and cyclin D2 protein were determined by western blotting. The total incubation time from the start of the TPA treatment to cell lysis is shown at the top of the panel. *β*-Actin was used as a loading control for western blotting.

We performed western blotting to investigate the expression of endogenous pro-HB-EGF, BCL6, and cyclin D2 in MKN45 cells. We found that MKN45 cells express endogenous pro-HB-EGF, BCL6, and cyclin D2 (data not shown). We determined whether the shedding of pro-HB-EGF could affect the expression of BCL6 and cyclin D2 protein levels. Cells were treated with 200 nM TPA for the indicated periods, after which the endogenous BCL6 and cyclin D2 expression levels were analysed by western blotting. In the absence of TPA, the level of cyclin D2 in MKN45 cells was low. However, after the TPA treatment, cyclin D2 expression level increased significantly, and BCL6 expression level was decreased (Figure 2A). Thus, BCL6 expression is decreased and, inversely, cyclin D2 expression is increased following pro-HB-EGF shedding in MKN45 cells.

### BCL6 degradation depends on HB-EGF-CTF nuclear translocation

To confirm that the degradation of BCL6 and the upregulation of cyclin D2 protein level depend on the production of HB-EGF-CTF, we used AG1478 and U0126. Before treatment with 200 nM TPA, for the purpose of EGFR signalling pathway inhibition, the cultured MKN45 cells were treated with 1 *μ*M AG1478 and 20 *μ*M U0126 for 60 min. Despite the complete inhibition of the EGFR signalling pathway (Figure 1B), BCL6 expression is decreased and cyclin D2 expression is increased after TPA treatment (Figure 2B). Then, we used metalloproteinase inhibitor KB-R7785 as an inhibitor of HB-EGF-CTF nuclear translocation by blocking the shedding of HB-EGF through inhibition of ADAM12 (Shimura *et al*, 2008). Before TPA stimuli, 10 *μ*M KB-R7785, with or without 1 *μ*MAG1478 and 20 *μ*M U0126, was added to the cells for 60 min to suppress the HB-EGF-CTF nuclear translocation. Despite the TPA treatment, BCL6 and cyclin D2 expression levels were unchanged regardless of the presence of inhibition of the EGFR signalling pathway (Figure 2C and D). These results indicate that the degradation of BCL6 and the upregulation of cyclin D2 depend on the production and nuclear translocation of HB-EGF-CTF after pro-HB-EGF shedding.

### BCL6 suppresses cyclin D2 expression in MKN45 cells

**Figure 3:**
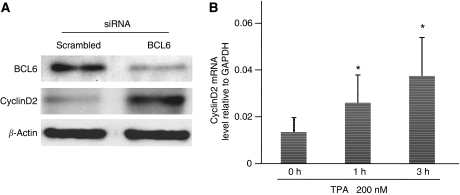
BCL6 suppresses cyclin D2 expression. (**A**) The levels of endogenous BCL6 and cyclin D2 protein were assessed by western blotting with anti-BCL6 and anti-cyclin D2 antibody, 48 h after transfection with siRNAs. (**B**) MKN45 cells were treated with 200 nM TPA for 1 or 3 h. The level of mRNA was analysed by real-time RT–PCR. The data are shown as means of three independent experiments. Bars, s.e. ^*^P<0.01 *vs* no TPA (0 h).

To determine if the upregulation of cyclin D2 protein is associated with BCL6, we transfected siRNA targeting BCL6 into MKN45 cells. The protein level of BCL6 in MKN45 cells was specifically reduced 48 h after transfection (Figure 3A). Cyclin D2 protein levels were not affected by transfection of the scrambled siRNA (Figures 2A and 3A). In BCL6 knockdown MKN45 cells, cyclin D2 protein level was increased, even in the absence of TPA, compared with untransfected cells (Figure 3A). We also evaluated cyclin D2 mRNA levels after TPA treatment to confirm that BCL6 regulates transcription of cyclin D2. Real-time RT–PCR data revealed that cyclin D2 mRNA levels were increased after TPA treatment (Figure 3B). These results indicate that BCL6 suppresses cyclin D2 expression in MKN 45 cells.

### Nuclear translocation of HB-EGF-CTF causes nuclear export of BCL6

**Figure 4:**
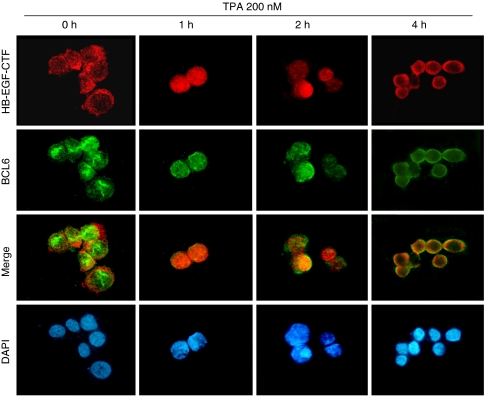
Nuclear export of BCL6 is detected following nuclear translocation of HB-EGF-CTF after TPA treatment. HB-EGF-CTF (red) and BCL6 (green) were visualised by immunofluorescent microscopy. Nuclei were stained blue by DAPI. MKN45 cells were treated with 200 nM TPA for 4 h. The total incubation time is shown at the top of the panel.

To investigate the behaviour of endogenous HB-EGF-CTF and BCL6 after TPA treatment, we performed immunostaining of HB-EGF-CTF and BCL6 in MKN45 cells. Before the TPA treatment, HB-EGF-CTF was predominantly localised in the cytoplasm, whereas BCL6 was observed in the nucleus. Fluorescent images revealed the translocation and accumulation of HB-EGF-CTF in the nucleus after 1 h of TPA treatment, whereas BCL6 remained in the nucleus (Figure 4). This result is consistent with previous data (Nanba *et al*, 2003). The nuclear export of BCL6 was detected after 2 h of TPA treatment (Figure 4), and both HB-EGF-CTF and BCL6 were predominantly localised in the cytoplasm after 4 h of TPA treatment (Figure 4). Thus, the nuclear export of BCL6 occurred after the nuclear translocation of HB-EGF-CTF was induced by ectodomain shedding.

### Interaction of BCL6 with HB-EGF-CTF increases after the HB-EGF-CTF nuclear translocation

**Figure 5:**
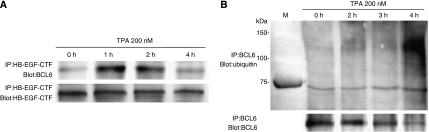
BCL6 interacts with HB-EGF-CTF and is degraded through the ubiquitin/proteasome pathway. (**A**) After treatment with 200 nM TPA for the indicated periods, HB-EGF-CTF was immunoprecipitated from an extract of MKN45 cells with anti-HB-EGF-CTF antibody and subsequently divided into two equal aliquots. One aliquot was subjected to western blotting with an anti-BCL6 antibody, and the second aliquot was subjected to western blotting with anti-HB-EGF-CTF antibody to control for loading. (**B**) After treatment with 200 nM TPA for the indicated periods, BCL6 was immunoprecipitated from an extract of MKN45 cells with anti-BCL6 antibody and subsequently divided into two equal aliquots. One aliquot was subjected to western blotting with an anti-ubiquitin antibody, and the second aliquot was subjected to western blotting with anti-BCL6 antibody to control for loading. M, size markers.

To analyse the amount of endogenous interaction between BCL6 and HB-EGF-CTF in MKN45 cells, we used antibodies against the cytoplasmic region of pro-HB-EGF. After cells were treated with 200 nM TPA for the indicated periods, BCL6 was precipitated from an extract of MKN45 cells by anti-HB-EGF-CTF antibody and then detected by western blotting with an anti-BCL6 antibody. Endogenous BCL6, which coimmunoprecipitated with HB-EGF-CTF, reached its maximum level after 1 h of TPA treatment (Figure 5A). These results, together with fluorescent images, indicate that nuclear BCL6 interacts with nuclear-translocated HB-EGF-CTF and that this interaction causes the nuclear export of BCL6.

### Ubiquitin/proteasome pathway mediates BCL6 degradation

To determine the mechanism for BCL6 degradation, we attempted to identify BCL6/ubiquitin conjugates. After treatment with 200 nM TPA for the indicated periods, cell lysates were collected and subjected to immunoprecipitation with anti-BCL6 antibodies. The immunoprecipitates were analysed by western blotting with anti-ubiquitin antibody (Figure 5B). Typical ladders representing multi-ubiquitinated forms of BCL6 were detected at high levels after 4 h of TPA treatment, suggesting that BCL6 degradation after the nuclear export of BCL6 is mediated by the ubiquitin/proteasome pathway.

### BCL6 expression in human normal gastric epithelium and other human tissues

**Figure 6:**
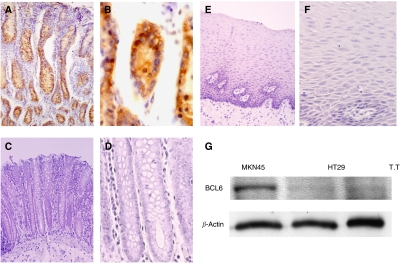
BCL6 expression in human normal gastric epithelium and other human tissues. (**A** and **B**) Immunohistochemical staining of human normal gastric epithelium. (**C** and **D**) Immunohistochemical staining of human normal colon epithelium (**E** and **F**). Immunohistochemical staining of human normal oesophageal epithelium. (Original magnification: A × 100; C and E × 200; B, D, and F × 400). (**G**) The level of endogenous BCL6 protein of MKN45 cells, along with HT29 cells (human colon cancer cell line) and T.T (human oesophageal cancer cell line), was analysed by western blotting for comparison.

We performed immunohistochemical staining of BCL6 in normal gastric tissues from humans. BCL6 was expressed in normal gastric epithelial cells, mainly in the bottom areas of gastric glandular ducts (Figure 6A and B). In addition, we examined BCL6 expression of other human tissues (Figure 6C–F). BCL6 was not expressed in normal mucosa of colon and oesophagus. We also evaluated endogenous BCL6 expression in human colon and oesophageal cancer cell lines by western blotting. These cancer cell lines also did not express endogenous BCL6 protein (Figure 6G).

### Immunohistochemical analysis of BCL6 in human gastric cancers

**Figure 7:**
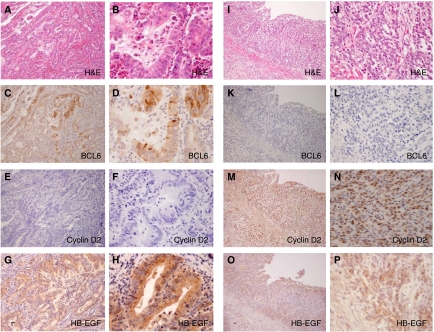
Immunohistochemistry of human gastric cancers. (**A**–**H**) Differentiated gastric cancer and (**I**–**P**) undifferentiated gastric cancer. (**A** and **B**) H&E staining; (**C** and **D**) BCL6 detected in the nucleus and cytoplasm of cancer cells; (**E** and **F**) cyclin D2 was negative; (**G** and **H**) HB-EGF was detected in the cytoplasm of cancer cells; (**I** and **J**) H&E staining; (**K** and **L**) BCL6 was negative; (**M** and **N**) cyclin D2 nuclear staining is observed in cancer cells; and (**O** and **P**) HB-EGF was detected in the cytoplasm of cancer cells. (Original magnification: **A**, **C**, **E**, **G**, **I**, **K**, **M**, **O** × 100; **B**, **D**, **F**, **H**, **J**, **L**, **N**, **P** **×**400).

The clinicopathological information from 100 cases of gastric cancers is summarised in Table 1. The study group consisted of 67 men and 33 women, with a mean age of 65.4 years (range, 37–88 years). There were 45 differentiated gastric cancers and 55 undifferentiated gastric cancers. Of the 100 cases of gastric cancer, 24 were BCL6 positive (positive rate=24%) and 76 cases were BCL6 negative. In BCL6-positive cells, both the nucleus and the cytoplasm were stained (Figure 7). There was a statistically significant difference in the histological type of BCL6-positive *vs* BCL6-negative cancers (*P*<0.05). BCL6 was highly expressed in differentiated cancers and was remarkably reduced or absent in undifferentiated cancers. There were no significant differences in gender, depth of invasion, and lymph node metastasis between the BCL6-positive and BCL6-negative groups (Table 1). In the Kaplan–Meier analysis, survival curves showed no significant differences between BCL6-positive and BCL6-negative cases (data not shown).

### Correlations between BCL6 and cyclin D2 expression

We analysed the relationship between BCL6 and cyclin D2 expression (Table 2). In the HB-EGF-positive group, statistically significant differences in cyclin D2 expression were noted in the BCL6-positive *vs* BCL6-negative subgroups (*P*<0.05) (Table 2A). Thus, BCL6 expression appears to be closely correlated with the downregulation of cyclin D2 expression. BCL6 was highly expressed in cyclin D2-negative cases and was remarkably reduced in cyclin D2-positive cancer cases in the HB-EGF-positive group. These results indicate that degradation of BCL6 might also induce cyclin D2 expression under the HB-EGF existence in human gastric cancer tissues as well as a gastric cancer cell line. In the HB-EGF-negative group, there were no significant differences in the relationship between BCL6 and cyclin D2 expression (Table 2B).

## Discussion

In the present study, we found that the transcriptional repressor BCL6 interacts with nuclear translocated HB-EGF-CTF after ectodomain shedding and that this interaction caused the nuclear export of BCL6. Cytoplasmic translocated BCL6 was degraded by the ubiquitin/proteasome pathway, and the degradation of BCL6 induced cyclin D2 expression in gastric cancer cell lines that expressed endogenous BCL6, HB-EGF-CTF, and cyclin D2. In the HB-EGF-positive group of human gastric cancer samples, BCL6 expression is also inversely correlated with cyclin D2 expression. We also identified a link between the expression of BCL6 (determined by immunohistochemistry) and the histological type of human gastric cancers.

12-0-Tetradecanoylphorbor-13-acetate has previously been demonstrated to induce cleavage of pro-HB-EGF by the activation of ADAM12 (Raab *et al*, 1994; Goishi *et al*, 1995; Asakura *et al*, 2002; Kurisaki *et al*, 2003; Higashiyama and Nanba, 2005). Our result revealed that TPA-dependent ectodomain shedding induced phosphorylation of both EGFR and MAPK. Treatment of MKN45 cells with TPA resulted in the degradation of BCL6 expression and the upregulation of cyclin D2 protein. Despite the suppression of EGFR signalling pathway, BCL6 protein level was decreased and cyclin D2 expression level was increased after TPA treatment. However, BCL6 degradation and cyclin D2 overexpression were inhibited by the suppression of the nuclear translocation of HB-EGF-CTF. Taking into account the interaction between BCL6 and cyclin D2, it was reported that the ZnF motifs of BCL6 bind to the cyclin D2 gene (Shaffer *et al*, 2000; Kinugasa *et al*, 2007). Fernandez de Mattos *et al* (2004) reported that BCL6 repressed cyclin D2 transcription through a STAT5/BCL6 site located within the cyclin D2 promoter. In addition, our data reveal that the knockdown of BCL6 increases the cyclin D2 protein level in MKN45 cells, even in the absence of TPA, compared with untransfected MKN45 cells and that cyclin D2 mRNA levels were increased after TPA treatment. The mechanism that BCL6 regulate transcription of cyclin D2 might also happen in gastric cancer cells. Considering these facts, our data indicate that HB-EGF-CTF nuclear translocation caused the degradation of BCL6 and that this degradation reverses cyclin D2 repression in gastric cancer cell lines that express endogenous BCL6, HB-EGF-CTF, and cyclin D2.

We used immunofluorescence microscopy to prove the nuclear export of BCL6, and we used immunoprecipitation to show the increased interaction of BCL6 with HB-EGF-CTF in MKN45 cells. Nanba *et al* (2003) reported that subsequent to proteolytic cleavage of pro-HB-EGF, HB-EGF-CTF was translocated from the cytoplasm into the nucleus, and this translocation triggered the nuclear export of PLZF. We proved a similar phenomenon in the case of BCL6. The translocation of HB-EGF-CTF from the cytoplasm into the nucleus and the nuclear export of BCL6 triggered by the translocation of HB-EGF-CTF were confirmed after TPA treatment. Immunoprecipitation revealed that the interaction of BCL6 with HB-EGF-CTF increased after HB-EGF-CTF nuclear translocation. These results indicate that nuclear BCL6 interacts with nuclear translocated HB-EGF-CTF and that this interaction causes the nuclear export of BCL6. Gene expression control by transcriptional repression occurs in the nucleus, and the nuclear export of BCL6 resulted in the loss of repressor function. Moreover, our data revealed that multi-ubiquitinated forms of BCL6 were increased after 4 h of TPA treatment, suggesting that the BCL6 degradation after the nuclear export of BCL6 is mediated by the ubiquitin/proteasome pathway. Niu *et al* (1998) also reported that BCL6 degradation was mediated by the ubiquitin/proteasome pathway. Considering these facts, our data clearly indicate that the nuclear export of BCL6 triggered by both the nuclear translocation of HB-EGF-CTF and the interaction of BCL6 with HB-EGF-CTF results in the deregulation of BCL6 transcriptional repressor function and the degradation of BCL6 through the ubiquitin/proteasome pathway. Taking the results of western blotting data into consideration, HB-EGF-CTF is translocated from the cytoplasm into the nucleus where it binds to BCL6. This interaction causes nuclear export and degradation of BCL6 and might result in the downregulation of BCL6 repressor activity for the cyclin D2 promoter in gastric cancer cells.

We also performed BCL6 immunohistochemistry on specimens of human gastric cancers. To our knowledge, this is the first report of an immunohistochemical analysis of BCL6 in human gastric cancers. BCL6 was considered to be produced at low levels in multiple tissues, and our immunohistochemical data revealed that BCL6 was expressed in normal gastric epithelial cells, mainly in the bottom areas of gastric glandular ducts. In the process of gastric cancer progression, BCL6 is decreased by HB-EGF-CTF signalling and the ubiquitin/proteasome pathway.

We found that BCL6 expression is closely linked with the downregulation of cyclin D2, as determined by immunohistochemistry, in cases of HB-EGF-positive human gastric cancer. Several studies have reported a correlation between decreased expression of cyclin D2 in cell lines and increased expression of the cell-cycle inhibitor p27^kip^ (Walz *et al*, 2006; Susaki *et al*, 2007). Taking into account the interaction between BCL6 and cyclin D2, it was reported that BCL6 repressed cyclin D2 transcription within the cyclin D2 promoter *in vitro* (Shaffer *et al*, 2000; Fernandez de Mattos *et al*, 2004; Kinugasa *et al*, 2007). In the present study, our immunohistochemical analysis revealed an inverse correlation between BCL6 and cyclin D2 expression in the group of HB-EGF-positive human gastric cancers. Thus, we found that BCL6 expression correlated with the downregulation of cyclin D2 expression in human gastric cancer tissues that are HB-EGF positive.

We also found that BCL6 expression was correlated with histological types. BCL6 was highly expressed in differentiated cancers and was remarkably reduced or absent in undifferentiated cancers. Previous studies of gastric cancers have found that genes such as *BUB1*, *eRF3/GSPT1*, and *hCG-1* were correlated with histological type (Grabsch *et al*, 2004; Malta-Vacas *et al*, 2005; Liu *et al*, 2007). However, the precise mechanism of association between the expression of these genes and the histology of gastric cancer is not well understood. There have been no previous reports of the relationship between BCL6 and gastric cancer histological type. Nitti *et al* (2002) reported that low p27^kip1^ protein expression, which is involved in cell-cycle control and apoptosis, is associated with poorly differentiated and advanced tumours. This data indicate that cell-cycle acceleration is associated with advanced undifferentiated cancers. Our present data reveal that BCL6 expression is reduced in undifferentiated gastric cancers. On the basis of these results, we speculate that in undifferentiated gastric cancers, the degradation of BCL6 induces cell-cycle acceleration through cyclin D2 expression and that this cell-cycle acceleration might be associated with undifferentiated gastric cancers. BCL6 expression levels in differentiated and undifferentiated gastric cancers may provide further evidence in gastric cancer progression.

In conclusion, this is the first report to investigate the transcriptional repressor BCL6 in gastric cancers. Our results indicate that the nuclear export and the degradation of BCL6 caused by the interaction with HB-EGF-CTF are apparently linked to cyclin D2 expression. These phenomena are shown in human gastric cancer cases as well as in gastric cancer cell lines. BCL6 expression is also associated with differentiated gastric cancers. Inhibition of HB-EGF-CTF nuclear translocation and maintenance of BCL6 function are important factors in the regulation of gastric cancer progression.

## Figures and Tables

**Table 1:** Clinicopathological factors and BCL6 expression in 100 gastric cancers

		**BCL6 expression**		
**Factor**	***n***	**Positive (score^a^)**	**Negative (score^a^)**	***P*-value**
*Gender*				
Male	67	17 (2.71±0.14)	50 (0.00±0.00)	0.646
Female	33	7 (2.43±0.29)	26 (0.00±0.00)	
				
*Histological type* b				
Differentiated	45	16 (2.82±0.10)	29 (0.00±0.00)	0.01
Undifferentiated	55	8 (2.25±0.31)	47 (0.00±0.00)	
				
*Depth of invasion*				
T1-2	40	12 (2.58±0.19)	28 (0.00±0.00)	0.3
T3-4	60	12 (2.67±0.18)	48 (0.00±0.00)	
				
*LN metastasis*				
Absence	25	6 (2.67±0.21)	19 (0.00±0.00)	1
Presence	75	18 (2.61±0.16)	57 (0.00±0.00)	
Age (years)				
(mean±s.d.)		65.6±10.7	65.2±12.0	

*n*=number of cases; LN=lymph node.

a The scores of each marker are average of ±standard error (s.e.).

b Classified based on structure of elements. ‘Differentiated’ includes tubular and papillary types, whereas ‘undifferentiated’ consists of signet-ring and poorly differentiated types.

**Table 2:** Relationship between BCL6 and cyclin D2

	**BCL6 (+)**	**BCL6 (−)**	**Total**	***P*-value**
*(A) HB-EGF* (+) *group*				
Cyclin D2 (+)	2	18	20	
Cyclin D2 (−)	11	17	28	0.02
	13	35	48	
				
*(B) HB-EGF* (−) *group*				
Cyclin D2 (+)	2	14	16	
Cyclin D2 (−)	9	27	36	0.3
	11	41	52	
